# Cognitive dysfunction in systemic lupus erythematosus is associated with disease activity and oxidative stress: a comparative study with rheumatoid arthritis for identifying biomarkers

**DOI:** 10.1186/s12868-023-00839-8

**Published:** 2023-12-13

**Authors:** Daniela Cabral de Sousa, Emmanuelle Silva Tavares Sobreira, Werbety Lucas Queiroz Feitosa, Ticiana Maria Pinto Moreira Aires, Leticia Pastuszka Paz Araújo, Andressa Laura Castro Silva, Caroline Brandão Joventino, Nathalia Maria Tomaz Silveira, Adriano José Maia Chaves-Filho, Danielle Silveira Macêdo, Pedro Braga-Neto

**Affiliations:** 1 Faculty of Medicine, University of Fortaleza (UNIFOR), Fortaleza, Ceará Brazil; 2 Department of Clinical Medicine, Faculty of Medicine, Federal University of Ceará, Fortaleza, Ceará Brazil; 3 Unichristus University Center, Fortaleza, Ceará Brazil; 4 Medical School Graduate Program, Faculty of Medicine, Federal University of Ceará, Fortaleza, Ceará Brazil; 5 Medical School Graduate Program, Faculty of Medicine, University of Fortaleza (UNIFOR), Fortaleza, Ceará Brazil; 6 Drug Research and Development Center, Department of Physiology and Pharmacology, Faculty of Medicine, Federal University of Ceara, Fortaleza, CE Brazil; 7 National Institute for Translational Medicine (INCT-TM, CNPq), Fortaleza, Ceará Brazil; 8 Center of Health Sciences, State University of Ceará (UECE), Fortaleza, Ceará Brazil

**Keywords:** Lupus, Cognitive dysfunction, Biomarkers, Rheumatoid arthritis

## Abstract

**Background:**

The prevalence and pathophysiological mechanisms of cognitive deficits (CD) Systemic Lupus Erythematosus (SLE) and Rheumatoid arthritis (RA) are very heterogeneous and poorly understood. We characterized CD in patients with SLE compared with RA patients and healthy controls. We compared the neuropsychological profile of SLE and RA with patients’ oxidative/inflammatory biomarkers for CD.

**Methods:**

We performed a cross-sectional study, including 50 SLE patients, 29 RA patients, and 32 healthy controls. SLEDAI and DAS28 assessed disease activity. SF-36 questionnaire and a battery of cognitive tests were applied to all participants. Blood samples were collected to determine IL-6, S100ß, myeloperoxidase (MPO), malondialdehyde and reduced glutathione (GSH) alterations.

**Results:**

In the SLE group, higher GSH was associated with the absence of CD (With CD = 69 ± 49, Without CD = 112 ± 81, p = 0.030), while higher IL-6 was associated with the presence of CD in the RA group (With CD = 603 ± 173, Without CD = 431 ± 162, p = 0.032). Regarding specific cognitive domains, in SLE higher MPO was associated with poor performance in reasoning and abstraction (p = 0.039), higher IL-6 was associated with poor performance in inhibitory control and attention (p = 0.031), and higher GSH was associated with better performance in memory(p = 0.021). Higher SLEDAI was associated with poor performance in semantic fluency(p = 0.031), inhibitory control, and attention in the SLE group(p = 0.037). In the RA group, higher DAS-28 was associated with poor performance in executive functions(p = 0.016) and phonemic fluency (p = 0.003).

**Conclusion:**

SLE patients’ disease activity, inflammatory state, and oxidative stress were associated with CD. In RA patients, CD was associated with disease activity and inflammatory state. These results encourage further studies with larger samples aiming to confirm oxidative stress parameters as biomarkers of CD in SLE patients.

**Supplementary Information:**

The online version contains supplementary material available at 10.1186/s12868-023-00839-8.

## Introduction

Systemic Lupus Erythematosus (SLE) is a chronic inflammatory autoimmune disease of unknown etiology, characterized by the involvement of multiple organs and systems [1]. Neurological and psychiatric symptoms are reported in 10 to 80% of patients before the diagnosis of SLE or during the disease course [2–4]. Cognitive dysfunction (CD) is SLE’s most common neuropsychiatric manifestation in 12 to 87% of patients. This large difference in the percentages of SLE patients showing neuropsychiatric manifestations observed in the studies reflects issues related to the study design, such as the populations studied, methodologies, and the difficulty in defining and evaluating cognitive dysfunction [5]. Several cognitive domains can be affected in patients with SLE [6].

The pathophysiological mechanisms of neuropsychiatric lupus (NPSLE) are very heterogeneous. However, proposed mechanisms include ischemic lesions caused by anti-phospholipid antibodies, immune complexes, complement activation, and inflammatory damage with increased blood-brain barrier(BBB) permeability, intrathecal autoantibodies, and other inflammatory mediators [7].Of note, oxidative stress (OS) is implicated in the pathogenesis of autoimmune diseases [8] and CD in Alzheimer’s disease [9], but, to date, there is no evidence of a similar association for CD in SLE. Despite the abovementioned results, currently, there are no definitive biomarkers for NPSLE diagnosis and treatment. Moreover, the recognition and diagnosis of NPSLE are very challenging as there is great heterogeneity of neurological symptoms and also the absence of standardized clinical evaluations [10].

Among the neuropsychiatric manifestations of SLE, cognitive dysfunction remains a major challenge. Its clinical evaluation is neither standardized nor routine, despite being a very common manifestation, and evidence is limited on the validity of screening instruments to assess CD in SLE. Furthermore, its multifactorial etiology makes the therapeutic approach difficult. ACR defined DC in LES as a significant deficit in any or all of the following cognitive domains: simple or complex attention, reasoning, executive skills, memory, visual-spatial processing, language, and psychomotor speed. Among these domains, attention, memory, and learning are most commonly affected. [11].

Although the mechanisms involved in the pathogenesis of SLE-related CD include the mechanisms already mentioned above for NPSLE, some of these mechanisms draw attention due to their relationship with the hippocampus and cerebral cortex. As an example, IL-6 can penetrate the BBB or be produced intrathecally; IL-6 mRNA is upregulated in the hippocampus and cerebral cortex of NPSLE patients [12].

Rheumatoid Arthritis (RA) is also an autoimmune disease associated with CD. The risk factors for CD in RA include cardiovascular, autoimmune, inflammatory alterations, hormonal changes, medication side effects, and psychiatric disorders [13]. However, despite previous studies showing inflammatory alterations as underlying mechanisms for CD in RA patients, there is no strong evidence for this association given mixed findings. A recent review also investigated the association of RA and Alzheimer’s disease. Although a few studies found a positive association between RA and cognitive impairment or dementia, there is a need to clarify possible shared mechanisms between them [14].

Considering the relevance and prevalence of CD in patients with autoimmune diseases and the lack of validated diagnostic biomarkers of these deficits in SLE and RA, we hypothesized that SLE and RA patients present impaired neuropsychological functioning associated with oxidative imbalance and pro-inflammatory alterations. Therefore, our primary outcome measure was to evaluate and compare the neuropsychological profile of SLE and RA patients. Our secondary outcome was obtaining these patients’ oxidative/inflammatory biomarkers for CD.

## Materials and methods

We performed a cross-sectional study at the rheumatic outpatient clinics of Hospital Universitário Walter Cantídio (HUWC) and the Integrated Medical Assistance Center of the University of Fortaleza (NAMI-UNIFOR), reference centers for rheumatic diseases in Fortaleza, Brazil. Data were collected in a single step from October 2018 to February 2020. Patients with SLE (SLE group) and RA (RA group) were consecutively evaluated based on their attendance at routine consultations at the respective services. Education and age-matched healthy individuals were selected as controls (Control group). The control group had no history of neurologic or psychiatric disease, and a screening test for anxiety and depression (Beck scale) was applied. Those who met the study criteria were invited to participate with informed consent.

For inclusion criteria, we considered participants from both sexes and diagnosis of SLE according to the SLICC criteria as inclusion criteria for the SLE group. We considered the diagnosis of RA according to EULAR/ACR criteria as inclusion for the RA group. All participants were 18–45 years old and agreed to participate in the study. As exclusion criteria, we considered: an education level of less than or equal to eight years, the presence of other diseases not related to SLE that could impact cognition, such as dementia, Parkinson’s disease, schizophrenia, obsessive-compulsive disorder, hypothyroidism, depression or anxiety disorder (diagnosed by a physician), as well as well as overlap with other autoimmune diseases such as Sjogren’s syndrome, scleroderma or inflammatory myopathies. This information was collected through participants’ self-reports and medical records. In the SLE group, previous neuropsychiatric manifestations were investigated, including stroke, seizures, encephalitis and others, and these conditions were not considered as exclusion criteria, as we chose to create a real-life cohort.

Sociodemographic, clinical, therapeutic, and habits data were collected from all patients. In addition, we aimed to obtain recent laboratory tests from medical records, such as blood count, urine summary, glucose, cholesterol rates, anti-DNA, C3, C4, and autoantibodies, but most of the patients lacked complete results.

The assessment of disease activity was performed using the SLE Disease Activity Index (SLEDAI) [15] for patients with SLE and the Disease Activity Score (DAS-28) [16] for patients with RA. The quality of life was assessed by the Medical Outcomes Study 36 – Item Short-Form Health Survey (SF-36) [17] The healthy controls had their sociodemographic, clinical, and habit data collected, and SF-36 measured quality of life.

All subjects underwent peripheral blood sample collection through venipuncture to evaluate inflammatory and oxidative biomarkers (IL-6, S100ß, myeloperoxidase - MPO, malondialdehyde -MDA, reduced glutathione - GSH). The blood was processed immediately after collection and stored at − 80 °C for a maximum period of 1 year, at the Neuropharmacology Laboratory at the Drug Research and Development Center (NPDM) of the Federal University of Ceará, where the analyses were carried out. S100ß and IL-6 plasma concentrations were performed by enzyme-linked immunosorbent assay (ELISA) technique according to the manufacturer’s manual with DuoSet® kits from R&D systems. Lipid peroxidation was determined in a spectrophotometer using a wavelength of 535 nm and expressed as micrograms of MDA/mL. The choice of biomarkers was based on the hypothesis that inflammatory mechanisms [18], breakdown of the blood-brain barrier and oxidative stress [8, 19] may be involved in the pathogenesis of NPSLE. Previous studies demonstrated a likely role of oxidative stress in the cognitive decline present in other clinical conditions [20, 21], but there is a lack of data regarding this association in autoimmune diseases.

A neuropsychologist applied the BR-SLE battery test (Brazilian adapted version) to assess the cognitive function of all subjects. The Supplemental Table 1 shows the battery tests, the corresponding cognitive domains evaluated by each test, and the bibliographic references. Here we better describe it, as follows:

- Colored Trails Test – adapted for the Brazilian population aged 18 to 86 and which assesses sustained and divided attention, perceptual tracking ability and graphomotor ability without suffering language interference due to the use of numbers and symbols [22].
- Stroop test adapted Victória version that assesses selective adaptation, mental flexibility and inhibitory control [22].
- Rey’s Complex Figure evaluates visual memory, visuo-spatial organization and planning with adaptation for the Brazilian population aged 5 to 88 years [23].
- RAVLT test that uses a list of simple and high-frequency words in Brazilian Portuguese to assess verbal episodic memory, validated and adapted for Brazil for the public aged 6 to 92 years [24].
- Verbal fluency (semantic and phonemic) task that assesses executive functions, semantic memory, storage of lexical content adapted for the adult Brazilian population [25, 26].
- WAIS-III subtests with Brazilian adaptation for adolescents from 16 years of age and adults up to 89 years of age: Matrix Reasoning that assesses visual information processing and abstract reasoning that is not interfered by language, Codes that evaluate processing speed, selective and concentrated attention, motor persistence and mental flexibility, and Number and Letters that aims to assess working and short-term memory [27].

The selection of these tests corresponds to the American College of Rheumatology (ACR) guidelines for cognitive assessment of SLE patients obtained from previous studies [28, 29]. All tests used were based on normative data adapted and validated for the Brazilian population, corresponding to age, sex and educational level. Individual scores were converted into standard scores. Therefore, a higher score on most tests means better performance in that cognitive domain. The exceptions are the Stroop (ST) and Color Trails Test (CTT) tests, which measure the time to perform a task; therefore, a lower score means better performance.

Despite the recommendation to use the battery of cognitive tests indicated by the ACR, there is no consensus in the literature on the definition of cognitive dysfunction in patients with SLE. In this way, we based ourselves on Alesi et al. [30] to define in our study the presence of Cognitive Impairment (CI) as a standard deviation equal to or less than − 2.0 (z score) in at least 03 cognitive domains.Then, we classified the three study groups as With CI or Without CI.

Data were collected and managed using the electronic data collection and management tool REDCap [31] hosted at the Clinical Research Unit of the UFC University Hospitals Complex. Data in numerical variables were presented as mean ± SD, while categorical variables were exposed in frequency and prevalence rate. The Mann-Whitney U test, Student’s t-test, ANOVA and Kruskal-Wallis were used to analyze the participants’ characteristics, verifying the non-adherence of the data to the Gaussian distribution. In addition, Pearson’s chi-squared test and Fisher’s exact test were used in investigating the association between categorical variables. A significance level of 5% was adopted, and all statistical analyzes were performed using the JAMOVI statistical program and Microsoft Excel 2016.

## Results

**Fig. 1:**
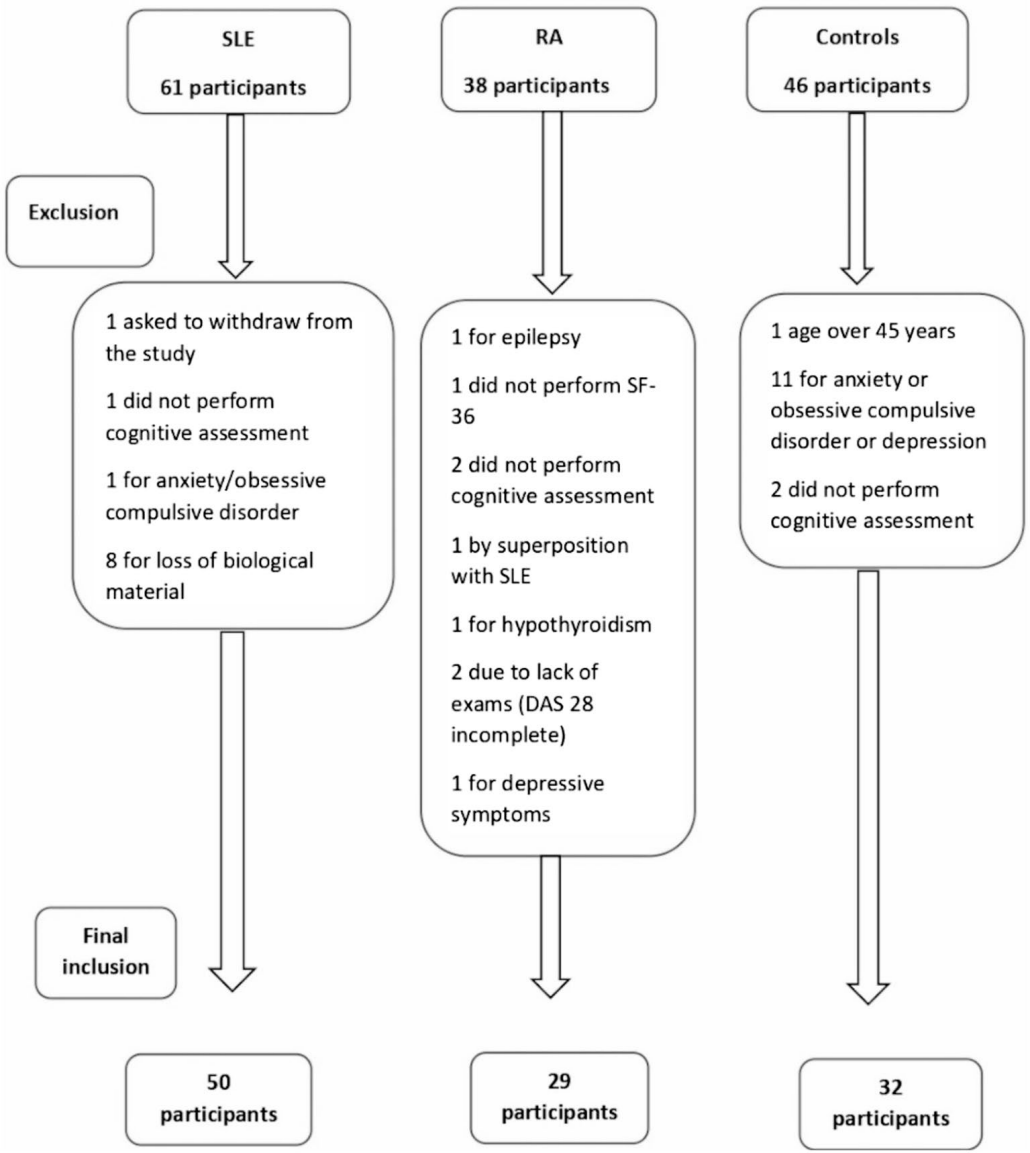
Selection flowchart and reasons for exclusion of study participants

Data were collected from 61 patients with SLE, 38 participants with RA, and 46 controls. However, during the protocol application, some participants added new clinical data or did not complete the protocol, thus being excluded from the study. The reasons for exclusion are shown in Fig. 1. In the end, 50 patients with SLE, 29 patients with RA, and 32 healthy controls were included in the study.

Demographic data for all participants are shown in Table 1, which describes data about gender, age, race, level of education of the three groups and disease duration in SLE and RA groups. There was no significant difference between the three groups regarding the distribution by gender, schooling or race among the three groups or in disease duration between the SLE and RA groups. However, there was a statistically significant difference in age between the groups and a post-hoc test found that the age in the AR group was significantly higher than the control group. There was no statistically significant difference in age between the SLE and Control groups. When we compared SLE and RA groups, we did not identify a statistically significant difference in terms of age or schooling.

**Table 1:** Demographic characteristics of participants and duration of disease in SLE and RA groups

	SLE	RA	Controls	p value
**Gender**				0.177c
Female	49 (98%)	28 (96.60%)	31 (96.9%)	
Male	1 (2%)	1(3.4%)	1 (3.1%)	
Age (years)	34 ± 7	37 ± 6	33 ± 6	0.029a
**Race**				0.564c
White	12(24%)	4(14.3%)	9(28.1%)	
Non-white	38 (76%)	25(85.7%)	23(71.9%)	
Level of education (years)	12.56 ± 2.24	13.16 ± 2.38	12.53 ± 2.02	0.404a
Disease duration	10.63 ± 6.72	8.22 ± 5.6		0.129a

Data expressed in n(%) and Mean ± Standard Deviation. a: Student’s t-test; c: Pearson’s Chi-squared test. SLE: Systemic Lupus Erythematosus, RA: Rheumatoid Arthritis

In the SLE group, we investigated previous neuropsychiatric manifestations through medical records. Of the 50 patients, 07 patients (14%) had previously demonstrated some neuropsychiatric manifestation, namely: 1 had presented pseudotumor cerebri, 1 myelitis, 2 seizures, 1 encephalomyelitis, 1 stroke and 1 central venous thrombosis. As no patient had presented recent neurological manifestations (all events had occurred more than 6 months ago), we chose not to exclude these patients.

Regarding the cardiovascular risk factors in the three groups, there was no significant difference in the frequency of systemic arterial hypertension (SAH) (SLE = 22%, RA = 31%, Control=,9,4%, p = 0.109), diabetes (SLE = 4%, RA = 6.9%, Control = 0%, p = 0.346), previous acute myocardial infarction (AMI) (no participant had this event), previous stroke (SLE = 2%, RA and Control = 0%, p = 0.540), smoking (SLE = 4%, RA = 0%, Control = 9.4%, p = 0.206), or alcohol use (p = 0.168) between groups. Regarding the frequency of dyslipidemia, there was a significant difference between groups (p = 0.000), as in the control group no individual had the condition, in contrast to the SLE group (12%) and the RA group (24.1%).

In Table 2, we show the comparison between the groups considering the classification with or without CI. There was no statistically significant difference between the three groups regarding the frequency of, but when we compare disease groups, the SLE group had a statically higher frequency of CI when compared to the RA group. There was no statistically significant difference between SLE and control group (p = 0.539) or between RA and control group.

**Table 2:** Frequency of Cognitive Impairment (CI) among the SLE, RA and control groups

	SLE(N = 50)	RA(N = 29)	Control(N = 32)	p value 0,089
With CI	30 (60%)	19 (34%)	17 (53%)	
Without CI	20 (40%)	19 (66%)	15 (47%)	

Data expressed in n(%). p value based on chi square test of independence. CI: cognitive impairment. SLE: Systemic Lupus Erythematosus, RA: Rheumatoid Arthritis

We analyzed the association between the presence of CI with age, education level, duration of disease, quality of life (measured by SF-36) and disease activity. There was no statistically significant difference in the three groups regarding age(With CI = 33 ± 7, Without CI = 35 ± 6, p = 0.196), SF-36 (With CI = 484 ± 181, Without CI = 515 ± 19, p = 0.271), activity scores (SLEDAI, p = 0.659; DAS-28, p = 0.089) and education level (With CI = 12.46 ± 1.90, Without CI = 12.97 ± 2.49, p = 0.311). However, regarding the disease duration in the RA group, a longer disease duration was associated with an absence of CI (With CI = 5.0 ± 4.2, without CI = 9.9 ± 5.6; p = 0.019).

When we analyzed specific cognitive domains in the SLE group, there was an association between higher education level and better performance in verbal memory (RAVLT rec, p = 0.026), visual constructive ability (RCF copy, p = 0.03), inhibitory control and selective attention (ST colors, p = 0.045; words, p = 0.025; and points, p = 0.014), processing speed (Codes, p = 0.01) and alternate attention (CTT1, p = 0.001). In both SLE and RA groups, we also found an association between better quality of life (SF-36) and better performance in alternate attention (LES group CTT2, p = 0.015; RA group CTT1, p = 0.012).

We also analyzed the association between CI and cardiovascular risk factors, like systemic arterial hypertension (SAH), diabetes, dyslipidemia, previous acute myocardial infarction (AMI), previous stroke, smoking, or alcohol use. There was no statistically significant difference regarding these variables in association with CI in the three groups.

Regarding the corticoid use and disease activity measures by SLEDAI and DAS-28 and the association with CI, we found no statistically significant difference in the SLE (SLEDAI, p = 0.659; corticoid use, p = 0.180) and RA groups (DAS28, p = 0.089; corticoid use, p = 0.064). In the SLE group, however, when we associate with specific cognitive domains, SLEDAI was negatively associated with semantic fluency (SVF, p = 0.031) and inhibitory control/selective attention (ST points, p = 0.032), meaning the higher score was correlated with poor performance on these tests. The same association occurred in the RA group regarding DAS-28 but with other cognitive domains: visual constructive ability (RCF copy, p = 0.016) and phonemic fluency (OVF, p = 0.003).

In Table 3, we described the comparison between the medium titers of possible biomarkers in SLE, RA and control groups. The SLE group had significantly lower MDA levels than RA participants In addition, MPO level was significantly higher in SLE than in Controls. However, there were no significant differences in IL-6, S100ß, and GSH levels between the three groups.

**Table 3:** Medium titers of possible biomarkers in the SLE, RA and control groups

Kruskal-Wallis (p)							
**Biomarkers**	**SLE**	**RA**	**Controls**	**All groups**	**SLE/RA**	**AR/** **Controls**	**SLE/Controls**
S100ß	395.88 ± 406.6 (223.5)	595.32 ± 1166.53 (218.16)	677.85 ± 1339.13 (223.5)	0.862			
IL-6	532.39 ± 205.61(341.88)	485.99 ± 182.01 (485.42)	595.24 ± 477.34(445.08)	0.612			
MDA	5.20 ± 5.92(4,15)	7.24 ± 4.92(6.8)	6.58 ± 4.47(6.54)	**0.010**	**0.016**	1,0	0.063
MPO	15.41 ± 18.96(8.16)	13.66 ± 17.28(5.02)	4.89 ± 5.86(1.57)	**0.013**	1,0	0.102	**0.014**
GSHr	88.48 ± 68.01(60.64)	94.74 ± 104.89(59.69)	79.15 ± 61.55(50.97)	0.50			

Data expressed in Mean ± Standard Deviation (Median). IL-6: -Interleukin 6, S100ß, MPO: myeloperoxidase, MDA: malondialdehyde, GSHr: reduced glutathione. Statistically significant p-values ​​are highlighted in bold

We analyzed the association of CD with biomarkers titles in the three groups, and the results are shown in Table 4. We found that GSH, a protective factor against oxidative stress, was positively associated with the absence of CD in the LES group. On the other hand, higher levels of IL-6 were associated with the presence of CD in the RA group.

**Table 4:** Association analysis between cognitive deficits (CD) and possible biomarkers in the SLE, RA and Control groups

		SLE			RA			Controls	
	With CD	Without CD	p value	With CD	Without CD	p value	With CD	Without CD	p value
IL-6	579 ± 153	475 ± 249	0.064	603 ± 173	431 ± 162	**0.032**	562 ± 524	633 ± 434	0.427
S100B	334 ± 290	472 ± 514	0.935	474 ± 681	653 ± 1,351	0.882	809 ± 1,563	529 ± 1,065	0.637
MDA	4.78 ± 2.49	5.70 ± 8.50	0.362	7.2 ± 8.1	7.3 ± 2.7	0.115	7.0 ± 3.0	6.2 ± 5.8	0.162
MPO	15 ± 22	16 ± 16	0.497	6 ± 6	17 ± 20	0.468	3.9 ± 5.1	6.0 ± 6.7	0.584
GSHr	69 ± 49	112 ± 81	**0.030**	89 ± 82	98 ± 116	0.730	83 ± 64	75 ± 61	0.720

CD: Cognitive deficits

Data expressed in Median ± Standard Deviation. p value based on Fisher’s exact test. Statistically significant p-values ​​are highlighted in bold

Finally, to analyze specific cognitive domains, we performed a partial correlation analysis, which were performed using the cognitive tests as outcome variables and probable biomarkers, activity scores (SLEDAI in the SLE group and DAS28 in the RA group) and corticosteroid use as independent variables. The partial correlation was adjusted for age, education and duration of illness (control variables). The choice of independent variables was based on the hypothesis that disease activity, biomarkers of inflammation and oxidative stress and the use of medications, especially corticosteroids, would be likely to influence the outcome. Whereas the outcome variables consisted of several tests, the independent variables consisted of 5 biomarkers, 2 activity scores (SLEDAI and DAS-28) and the use of corticosteroids, and it has generated a large amount of data, we chose to describe above only those results that were statistically relevant.

In Table 5, the results of the partial correlation analysis in the SLE group. We observed that a higher SLEDAI in the SLE group was associated with poor performance in ST points (Inhibitory control and selective attention, p = 0.037), and higher IL-6 levels were associated with poor performance in ST words (p = 0.031). Higher MPO activity was associated with poor performance in matrix reasoning (logical reasoning and abstraction, p = 0.039). GSH, an endogenous antioxidant, was positively associated with better performance in RAVLT B (verbal episodic memory, p = 0.021). Corticosteroid use was associated with poor performance in RAVLT B (p = 0.003), RCF copy (constructive praxis, planning, and visuospatial organization, p = 0.033), and ST colors (p = 0.047). Conversely, MDA activity was positively associated with better performance in CTT1 (alternating attention, p = 0.011). The other parameters evaluated did not reach statistical significance.

**Table 5:** Partial correlation analysis between cognitive tests and disease activity (SLEDAI), biomarkers (S100ß, IL-6, MDA, MPO, GSHr) and corticosteroid use in the SLE group, adjusted for age, education and disease duration

Cognitive tests	Cognitive domain	S100B	IL-6	MDA	MPO	GSHr	SLEDAI		Corticoid use
RAVLT B	Verbal memory	0.028(0.875)	0.316(0.065)	0.016(0.929)	0.211(0.225)	**0.390(0.021)** **Better performance**	0.027(0.878)		**-0.491(0.003)** **Poor performance**
RCF copy	Visual constructive ability	0.005(0.979)	-0.103(0.555)	0.174(0.317)	-0.028(0.873)	0.0(0.998)		-0.099(0.570)	**-0.361(0.033)** **Poor performance**
ST points	Inhibitory control and selective attention	-0.047(0.789)	0.045(0.799)	-0.136(0.437)	0.042(0.813)	0.0(0.999)	**0.354(0.037)** **Poor performance**		0.023(0.894)
ST words	Inhibitory control and selective attention	-0.011(0.951)	**0.365(0.031)** **Poor performance**	-0.131(0.452)	-0.043(0.806)	0.035(0.843)	0.224(0.195)		0.063(0.721)
ST colors	Inhibitory control and selective attention	-0.214(0.218)	0.142(0.417)	-0.268(0.120)	-0.093(0.595)	0.024(0.890)	0.173(0.321)		**0.339(0.047)** **Poor performance**
Matrix reasoning	Reasoning	-0.199(0.253)	-0.206(0.236)	0.176(0.313)	**-0.350(0.039)** **Poor performance**	0.069(0.695)	0.156(0.371)		-0.272(0.113)
CTT1	Attention Processing speed	-0.069(0.694)	0.319(0.062)	**-0.425(0.011)** **Better performance**	-0.166(0.340)	-0.047(0.787)	0.159(0.362)		0.234(0.177)

Data expressed as cc: correlation coefficient, p:p value. Statistically significant p-values ​​are highlighted in bold

Using the same analysis, disease activity (DAS-28) in the RA group was associated with poor performance in phonemic fluency (OVF, p = 0.036), and corticosteroid use was associated with better performance in memory from verbal clue (RAVLT rec, p = 0.030). However, the other parameters evaluated did not reach statistical significance.

## Discussion

Cognitive dysfunction is always challenging in the follow-up of patients with autoimmune diseases since cognitive functions are essential for daily activities. Unfortunately, despite being a common clinical manifestation, it is generally not systematically addressed in these patients and influenced by various factors.

In the present study, we observed significant associations between inflammatory/oxidative markers with cognitive deficits, notably higher levels of GSH associated with better cognitive performance in patients with SLE, and higher levels of IL-6 associated with worse cognitive performance in patients with RA. These findings support our hypothesis that impaired neuropsychological functioning in SLE and RA may be associated with oxidative imbalance and pro-inflammatory alterations. Oxidative stress damages the function and structure of the brain structure including areas related to cognition. Indeed, GSH reduces oxidative stress by eliminating H2O2 and previous studies have already described that GSH may be a potential protective biomarker against neurodegenerative disease like Alzheimer’s dementia and cognitive decline [32]. IL-6 was also described to be negatively correlated to cognitive performance. Its mechanism is not well understood, but may include interaction with vascular,,neurodegenerative processes or even a direct neurotoxic action. Recent findings also support the idea that target pro-inflammatory IL-6 signaling may be a strategy to alleviate memory impairment in patients with Alzheimer’s disease [33].

Although we did not identify an association between a broader cognitive impairment (according to the criteria described, involving at least three domains) and disease activity, in secondary data we found associations between SLEDAI and DAS-28 and deficits in some specific cognitive domains, such as executive functions and fluency. Besides, in a partial correlation analysis, we found that greater disease activity, inflammation, and oxidative alterations in SLE patients were associated with poor performance in specific cognitive domains. It is worth mentioning that in our sample, MPO activity was significantly higher in the SLE than in the control group. Although MDA levels were positively correlated with better performance in alternating attention, this parameter in the SLE group was lower than in the RA group. Therefore, this result may reflect the low sensitivity of the MDA test for determining lipid peroxidation in SLE in our sample.

We also observed that SLE patients using corticosteroids performed poorly in some cognitive domains. Ouanes et al. [34] reviewed the relationship between cortisol and CD in Alzheimer’s disease. These authors concluded that elevated cortisol levels were associated with poorer overall cognitive functioning. Indeed, cortisol is neurotoxic for hippocampal neurons, promoting oxidative stress and amyloid β peptide toxicity.

In the present study, we did not find any associations between the variables of interest (disease activity and oxidative alterations) and the overall primary outcome measure of cognitive dysfunction, but we found associations with specific cognitive domains of interest, such as executive function, verbal fluency and memory. These cognitive functions are commonly affected in SLE according to previous studies [35]. It would be interesting if new studies with a larger sample size could better evaluate these associations.

In addition, we found an association between the absence of CD and a longer disease duration in the RA group. Therefore, we hypothesized that longer disease duration could be linked with better disease control. Besides, in this group, there was an association between CD and higher levels of IL-6. The partial correlation analysis showed that disease activity (DAS-28) was associated with poor phonemic fluency performance. In line with these findings, Katchamart et al. [36] developed a multicenter study with 464 patients with RA and demonstrated that high cumulative RA disease activity is associated with CD. Therefore, inflammatory status and disease activity could be associated with cognitive symptoms in RA.

Previous studies have attempted to find biomarkers for neuropsychological alterations, including CD, in SLE patients. To this end, cytokines were largely studied since they function as neuromodulators and inflammatory mediators. Hirohata et al. [37], by evaluating the pathogenesis of NPSLE, found that IL-6 serum and CSF levels were significantly elevated in the acute confusional state (ACS) compared with non-ACS diffuse NPSLE (including CD) or focal NPSLE. Kozora et al. [38] found no relationship between increased serum IL-6 levels and cognitive impairment in SLE patients without overt neuropsychiatric symptoms. Finally, a review article [39] reported that although serum IL-6 concentrations were significantly increased in SLE patients during acute phases, measurements of serum IL-6 levels in stable disease conditions yielded mixed results. These authors concluded that as serum IL-6 is not always increased in SLE patients, disease activity (i.e., acute attacks versus chronic stable conditions) should also be considered when assessing the role of peripheral IL-6 in CD. Our results corroborate this hypothesis since we observed that in partial correlation analysis, elevated SLEDAI scores and IL-6 levels were associated with cognitive impairments in the same domains.

Regarding our findings of an association between levels of IL-6 and CD in RA patients, we recently published a systematic review article [40] on biomarkers of cognitive dysfunction in RA, and IL6, along with other cytokines (IL-2, IL-4 and tumor necrosis factor α),negatively correlated with memory and positively correlated with executive functions. This study draws attention to the scarcity of data in the literature on this subject in RA population. A possible explanation for this association is that high levels of IL-6 in the cerebrospinal fluid derived from astrocytes may lead to disruption of the brain-cerebrospinal fluid barrier, most notably around the hypothalamus, which might result in inflammatory activation of microglia to damage the hypothalamic neurons and impaired cognitive function [41]. Yang and coworkers [42] also reinforced the role of IL-6 and other cytokines like IL-1β and TNFα in disruption of blood–brain barrier (BBB), leading to neurological degeneration in such diseases as Alzheimer’s disease (AD), stroke, multiple sclerosis (MS), and posttraumatic brain injury (TBI).

The association between disease activity and cognitive dysfunction in SLE is a very controversial subject in the literature, with studies finding divergent results. Raghunath and coworkers [43] evaluated the cognitive function of 89 patients with SLE in a cross-sectional study, using a battery of neuropsychological tests. This study found a correlation between cognitive dysfunction and organ damage, but no correlation with disease activity. Other studies with larger samples also did not identify this correlation [44]. Conversely,a cross-sectional study similar to the present study, using batteries of cognitive tests, identified a correlation between CD and disease activity [45].

An interesting longitudinal study by Ceccarelli and coworkers evaluated 43 patients with SLE for cognitive dysfunction over 10 years, and observed a reduction in cognitive decline from 20.9 to 13.9% [46]. The authors hypothesize that improvements in the therapeutic approach to SLE over time led to these results. Although this study did not identify a specific correlation between disease activity and cognitive dysfunction, we can assume that throughout the course of the disease, better control of the disease seems to be associated with less cognitive dysfunction.

A few studies have associated serum levels of S100β with CD in SLE and RA patients. However, we did not find such an association in the present study. Lapa et al. [47] found an association between high S100β levels and cognitive deficits in SLE children. Baptista et al. [48] demonstrated an association between high S100β levels and cognitive decline in patients with active RA. However, another recent study evaluating CD in SLE patients did not identify an association with S100β levels [49]. Cognitive disorders in RA are less frequent than SLE (around 30%) and still have debatable pathophysiological mechanisms. There are few studies evaluating this protein in SLE and RA patients, and a conclusion about its real mechanism in patients with autoimmune diseases is still lacking.

Since we found associations between oxidative stress markers (MPO and GSH) and CD, it is essential to note that such an association was not previously observed in autoimmune diseases. On the other hand, in neurodegenerative diseases such as Alzheimer’s, increased production of reactive oxygen species (ROS) can affect neurons’ synaptic activity and neurotransmission, leading to CD [9]. Besides that, an oxidative imbalance [excess production of ROS and reactive nitrogen species (RNS)] associated with an inflammatory response is observed in many autoimmune diseases [8], leading to tissue damage.

Hassan et al. [50] pointed out that increased ROS levels in RA patients lead to a pro-oxidant environment, i.e., decreased antioxidant activity and increased MDA levels, resulting in lipid peroxidation and consequent cell damage. This pro-oxidant environment is one possible underlying mechanism of SLE disease activity. Unlike our results, this previous study found higher MDA levels in RA patients compared to SLE and control subjects, while we found higher MDA in RA compared to SLE patients. Hassan et al. [50] also found that MDA levels in SLE patients were correlated with alopecia and nephritis, but they did not mention neurological symptoms. Telles et al. [51] showed that MPO activity was associated with articular manifestations in SLE patients. To our knowledge, the results of the present study are the first to demonstrate an association between an oxidative stress imbalance and CD in SLE patients.

Corroborating previous findings, we also observed that better quality of life (measured by the SF-36) led to better performance on attention tests in both groups of patients. In addition, a systematic review of CD and quality of life in SLE [52] concluded that cognitive impairment negatively correlates with the quality of life and social life participation in this population.

Regarding education, cognitive performance in the SLE group was positively associated with higher education in several domains, but this correlation did not occur in the RA group. The impact of education level on cognitive functioning in the general population is well known [53]. Papastefanakis et al. [54] analyzed 71 SLE patients and showed that screening for cognitive impairment by Montreal Cognitive Assessment (MoCA) was significantly affected by education level. In addition, MoCA was positively correlated with an extensive neuropsychological test battery in this study, corroborating the results.

In the present study, we have also analyzed cardiovascular risk factors because they are closely correlated with ischemic brain injuries and could consequently be a confounding factor in the analysis of cognitive dysfunction in our patients. However, in our sample, these factors did not correlate with any of the variables of interest assessed.

The higher mean age of the RA group should be considered a limitation of this study, and age is known to have a big impact on cognitive test performance. This find can be partially explained by the epidemiology of RA, whose age group is 30–50. Despite this, we corrected age when analyzing the results through partial correlation, and only disease activity was significantly associated with cognitive impairment in this group. Furthermore, considering that the average age of the RA group was 37 years and the maximum age was 45 years, we believe that this may minimize the impact of the age factor on the interpretation of findings of cognitive dysfunction in the present sample.

Another limitation was the small sample, particularly in the RA and control groups. Nevertheless, using a battery of cognitive tests instead of screening tests led to greater reliability in the cognitive assessment. By performing this battery of tests, we could identify the most affected cognitive domains and associate them with the diseases and inflammatory/oxidative alterations. Despite these limitations, our results reinforced the impact of quality of life and education on cognition.

Surprisingly, we found a higher incidence of CD in the healthy control group compared to the RA group. As patients were screened for other pathologies that could interfere with cognition and for symptoms of anxiety and depression, we postulate that perhaps other factors had an impact on the poor performance of the control group in cognitive tests. A possible explanation for this finding would be that the selection of control group was mostly during their working hours. The lack of attention or the rush to complete the neuropsychological evaluation and return to their activities may have impacted data collection. This aspect limited its comparability with patient groups. However, the main results of our study were identified when we analyzed the groups of patients separately, especially in the SLE group, with the largest number of participants. At the same time, we were able to demonstrate that cognitive dysfunction in the SLE group was significantly greater in the SLE group compared to the RA group, despite the SLE patients being younger, and this finding is consistent with the literature.

In conclusion, the present study showed that disease activity and pro-inflammatory/oxidative imbalances are associated with CD in SLE patients. The results in RA showed an association between CD and inflammatory status. In clinical practice, this means that patients with SLE or active RA should probably be screened for CI by a more detailed neuropsychological evaluation. Oxidative stress markers and IL-6 levels are predictive markers of CD in SLE and RA. In this regard, future studies with larger sample sizes must be designed to increase the level of evidence for this finding. Additionally, longitudinal studies are needed to improve the evaluation of inflammatory/oxidative biomarkers in SLE and RA.

### Electronic supplementary material

Below is the link to the electronic supplementary material.

**Supplementary Material 1**: Supplemental table 1 – Cognitive domains and BR-SLE battery tests

## Data Availability

All materials and data used in this study will be made available upon request. All of the material is owned by the authors and no permissions are required. All of the material are available for publication. To consult and check the data of this study, contact pbraganeto@ufc.br or danicsousa12@gmail.com. All of the material is owned by the authors and no permissions are required.

## List of Abbreviations

ACR: American College of Rheumatology
ACS: Acute confusional state
AMI: Acute Myocardial infarction
Anti-NR2: Antibodies against the NR2 subunit of N-methyl D-aspartate receptor
BDNF: Neurotrophin brain-derived neurotrophic factor
CD: Cognitive deficits
CTT: Color Trails Test
DAS: Disease Activity Score
ELISA: Enzyme-linked immunosorbent assay
GSH: Reduced glutathione
HUWC: Hospital Universit?rio Walter Cant?dio
MDA: Malondialdehyde
MoCA: Montreal Cognitive Assessment
MPO: Myeloperoxidase
NAMI-UNIFOR: Integrated Medical Assistance Center of the University of Fortaleza
NPDM: Neuropharmacology Laboratory at the Drug Research and Development Center
NPSLE: Neuropsychiatric lupus
OS: Oxidative stress
RA: Rheumatoid arthritis
RNS: Reactive nitrogen species
ROS: Reactive oxygen species
SAH: Systemic arterial hypertension
SF-36: Medical Outcomes Study 36 ? Item Short-Form Health Survey
SLE: Systemic Lupus Erythematosus
SLEDAI: SLE Disease Activity Index
