# Supplementary material for: Cognitive dysfunction in systemic lupus erythematosus is associated with disease activity and oxidative stress: a comparative study with rheumatoid arthritis for identifying biomarkers
Source: BMC Neurosci. 2023 Dec 13;24:66. doi: 10.1186/s12868-023-00839-8 (PMC10717202; doi:10.1186/s12868-023-00839-8)
Supplement: Supplementary file 1 — Supplementary Material 1: Supplemental table 1 – Cognitive domains and BR-SLE battery tests [file 12868_2023_839_MOESM1_ESM.docx]

**Supplemental table 1– Cognitive domains and BR-SLE battery tests**

| **Cognitive domains** | **Tests** | **Standardization for the Brazilian population** |
| --- | --- | --- |
| **Reasoning** | Matrix reasoning | Nascimento (2004)²⁷ |
| **Processing speed** | Codes | Nascimento (2004)²⁷ |
| **Working memory** | SLN | Nascimento (2004) ²⁷ |
| **Attention/Processing speed** | CTT 1 and 2 | Campanholo (2014)²² |
| **Visual constructive ability** | RCF copy | Oliveira (1999)²³ |
| **Phonemic verbal fluency** | SVF | Brucki (2004)²⁶ |
| **Semantic verbal fluency**  **Inhibitory control and selective attention** | OVF    ST (colors, words and points) | Machado (2009) ²⁵    Campanholo(2014)²² |
| **Verbal memory** | RAVLT (total, B and recordation) | Magalhães (2010) ²⁴ |
| **Visual memory** | RCF evocation | Oliveira (1999)²³ |

BR-SLE: Brazilian Systemic Lupus Erythematosus; SNL: Sequence of Numbers and Letters; CTT: Color Trails Test; RCF: Rey’s Complex Figures; SVF: Semantic Verbal Fluency; OVF: Orthographic Verbal Fluency; RAVLT: Rey Auditory Verbal Learning Test; ST: Stroop test. BR - Brazilian adapted version
